# Encapsulated polycaprolactone with triazole derivatives and selenium nanoparticles as promising antiproliferative and anticancer agents

**DOI:** 10.5599/admet.1789

**Published:** 2023-06-28

**Authors:** Ahmed E. Abdelhamid, Ahmed A. El-Sayed, Samira A. Swelam, Abdelmohsen M. Soliman, Ahmed M. Khalil

**Affiliations:** 1 Polymers & Pigments Department, National Research Centre, El-Bohouth St., Dokki - 12622, Giza, Egypt; 2 Photochemistry Department, National Research Centre, 33 El-Bohouth St., Dokki - 12622, Giza, Egypt; 3 Therapeutic Chemistry Department, National Research Centre, 33 El-Bohouth St., Dokki - 12622, Giza, Egypt

**Keywords:** Carbohydrate polymers, metal nanoparticles, breast cancer cell line (MCF7), murine fibroblast normal cell line (BALB/3T3)

## Abstract

**Background and purpose:**

Polycaprolactone nanocapsules incorporated with triazole derivatives in the presence and absence of selenium nanoparticles were prepared and evaluated as antiproliferative and anticancer agents. Polycaprolactone nanoparticles were prepared using the emulsion technique.

**Experimental approach:**

The prepared capsules were characterized using FT-IR, TEM and DLS measurements. The synthesized triazolopyrimidine derivative in the presence and absence of selenium nanoparticles encapsulated in polycaprolactone was tested for its in vitro antiproliferative efficiency towards human breast cancer cell line (MCF7) and murine fibroblast normal cell line (BALB/3T3) in comparison to doxorubicin as a standard anticancer drug.

**Key results:**

The results indicated that encapsulated polycaprolactone with selenium nanoparticles (SeNPs) and triazole-SeNPs were the most potent samples against the tested breast cancer cell line (MCF7). On the other hand, all compounds showed weak or moderate activities towards the tested murine fibroblast normal cell line (BALB/3T3).

**Conclusion:**

As the safety index (SI) was higher than 1.0, it expanded the way for newly synthesized compounds to express antiproliferative efficacy against tumour cells. Hence, these compounds may be considered promising ones. However, they should be examined through further in-vivo and pharmacokinetic studies.

## Introduction

With advances in drug discovery technology, more than 40 % of newly discovered drug candidates have low aqueous solubility that limits their administration roots [1]. The encapsulation technique is considered an efficient alternative to overcome the solubility difficulty and enable the delivery of these compounds into purposed sites in the body. The capsules (micro or nanocapsules) are made up of two parts: the core and the shell material. In general, the core material contains an active ingredient, whereas the shell material protects the core material from the outside environment and allows for good release characteristics. Micro or nanocapsules are very useful carriers due to their physiological stability and ability to reach local areas by targeting intended tissues or organs. Natural and some synthetic polymers are used as encapsulating materials due to their biodegradability and excellent biocompatibility. Polycaprolactone (PCL) is widely used for drug delivery and other medical applications with stable properties over time. It is unaffected by pH in aqueous solution [2-4]. Polycaprolactone (PCL), either combined with or without poly(ethylene glycol) nanoparticles (NP), was synthesized and assessed as potential drug nanocarriers[5]. The investigated NPs did neither affect the cells' viability nor elicit an immune response in the RAW 264.7 mouse murine macrophage cell line. Preparation of paclitaxel-loaded polycaprolactone nanoparticles by nanoprecipitation was investigated for lung cancer treatment [6]. The prepared nanoparticles were coated with chitosan or poly-l-lysine to obtain a cationic surface charge and to increase the cellular interaction. The in vitro release studies showed a prolonged release for up to 96 hours. However, less than 50 % of the therapeutic load was released in the first 24 hours. The medication's core material can be used to treat several diseases such as angina, hypertension, Raynaud's phenomenon, and cancer, *etc*. [7–9]. Heterocyclic compounds are well known for their high biological activity, including anticancer capability [10,11]. Triazole derivatives isolated from natural products or synthesized in laboratory conditions possess diverse pharmacological properties such as antibacterial, antitubercular, anticancer, and antimalarial activities [12-16]. The most important tools in organic transformations and pharmaceuticals are multicomponent reactions. Triazole derivatives based on reacting hydrazinylthienopyrimidine with a number of monosaccharides for antiviral activity against influenza virus H5N1 were tested [15]. Moreover, selenium (Se) is a substantial nutrient for humans. In food, it can be obtained from various sources, including meats and fish. Selenium nanoparticles (SeNPs) (the zero-valent selenium) have recently drawn more scientific attention. This characteristic may be correlated to their obvious biological activities and biosafety properties.SeNPs have a variety of biomedical applications, including drug and targeted gene delivery, anticancer, antibacterial and anti-inflammatory activity, and biosensors [17,18]. Many reports describe the synthesis of SeNPs using various methods such as laser ablation, microwave-assisted synthesis, chemical reduction, electrodeposition, solvothermal synthesis, and green synthesis. However, harsh chemical conditions such as acidic pH and high temperatures limit their use in biomedical applications [19-21]. Cancer is one of the most important modern health issues because it knows no boundaries and can affect any organ. The World Health Organization (WHO) estimates that cancer is among the major causes of roughly many million deaths worldwide [22]. The majority of anticancer medications created to treat cancer have been demonstrated to cause cancer cells to undergo apoptosis [23]. Although there are various treatments available, persistent resistance to these treatments focuses on the need for innovative cancer control alternatives [24].

We aim in this study to evaluate the *in vitro* antiproliferative potency of polycaprolactone nanoparticles encapsulated with triazole derivative and conjugated with SeNPs. There are four formulations prepared in this study; blank polycaprolactone **1**, polycaprolactone encapsulated with triazole derivative **2**, polycaprolactone encapsulated with triazole-SeNPs **3**, polycaprolactone encapsulated with SeNPs **4.** The bioactivity assessment of these formulations towards human breast cancer (MCF7) and Murine fibroblast normal (BALB/3T3) cell lines will be investigated.

## Experimental

### Chemistry

Stuart melting point apparatus (SMP 30) was used to measure melting points. The reactions were monitored using pre-coated (0.25 mm) silica gel plates (Merck 60 F_254_, Germany). The spots were visualized using a UV lamp (254 nm). NMR spectra were recorded in (DMSO) at ^1^H NMR (400 MHz) and ^13^C NMR (100 MHz) using TMS as an internal standard on a Bruker NMR spectrometer. Mass spectra were performed on the direct inlet part of the mass analyzer in a Thermo Scientific GCMS model ISQ. The UV-Vis (Shimadzu spectrophotometer) was used to monitor the formation of selenium nanoparticles in the range of 400 and 700 nm. The shape and size of SeNPs were practically obtained using high-resolution transmission electron microscopy (HRTEM) JEOL (JEM-2100 TEM). Their diameters were assessed by using ImageJ software. Specimens for TEM measurements were prepared by placing a drop of colloidal solution on a 400 mesh carbon-coated copper grid with evaporating the solvent in the air at 25 °C. Dynamic light scattering was used to determine the average diameter and size distribution of the encapsulated samples using Zeta Sizer (Nano-ZS, Malvern Instruments Ltd., Zeta sizer Ver, 704, UK). The samples were sonicated for 30-60 minutes before the assessment to obtain a good suspension of the particles in the solution.

### Ethyl-7-acetamido-5-(5-methylfuran-2-yl)-[1,2,4]triazolo[1,5-a]pyrimidine-6-carboxylate (2)

A mixture of compound **1** (2.87g, 10 mmol) and acetic anhydride (10 mL) was heated under reflux for 4 h. The reaction mixture was allowed to cool to room temperature and poured into water (100 mL). The solid formed was collected by filtration, dried, and crystallized from ethanol.

Brown crystals; yield 60 %; mp 120-122 °C, IR (KBr, *ν* /cm^-1^); 3436 (NH), 1711, 1659 (C=O), 1054 (C=C); ^1^H-NMR (DMSO-*d_6_*, *δ* / ppm), 1.20-1.28 (t, 3H, *J*=5.4Hz, CH_3_), 2.33 (s, 3H, CH_3_), 2.47 (s, 3H,CH_3_), 4.22-4.28 (q, 2H, *J* = 5.4Hz, CH_2_), 6.50-6.55 (d, 1H, *J* = 2.5 Hz, -CH, furan ring), 7.43-7.45 (d, 1H, *J* = 2.5 Hz, -CH, furan ring), 7.99 (s, 1H,CH, triazole proton), 10.75 (broad singlet (br s), 1H, NH, D_2_O exchangeable); ^13^C-NMR (DMSO-*d_6_*, *δ* / ppm), 21.46, 22.62, 23.88, 62.40, 95.04, 112.03, 116.05, 138.96, 144.79, 147.51, 157.84, 161.21, 163.05, 168.01, 172.49; MS ((*m/z*) / %) (329, 21); Analyatical calculated for C_15_H_15_N_5_O_4_ (329.32), C, 54.71; H, 4.59; N, 21.27; Found: C, 54.60; H, 4.66; N, 21.19 %.

### 7-amino-8-methyl-5-(5-methylfuran-2-yl)pyrimido[5,4-e][1,2,4]triazolo[1,5-a]pyrimidin-6 (7H)-one (3).

The equimolar amount of compound **2** (3.29, 10 mmol) and hydrazine hydrate (10 mmol) in ethanol (20 mL) was refluxed for 6 h. The precipitate formed was filtered off, washed with cold ethanol, dried and recrystallized from ethanol.

Brown crystals, yield 60 %; mp 220-222 °C, IR (KBr, *ν* / cm^-1^); 3430 (NH_2_), 1669 (C=O), 1054 (C=C); ^1^H-NMR (DMSO-*d_6_*, *δ* / ppm): 2.12 (s, 3H, *-*CH_3_), 2.33 (s, 3H, -CH_3_), 3.63 (br s, 2H, -NH_2_ exchangeable with D_2_O), 6.31-6.37 (d, 1H, *J* = 2.5Hz, -CH, furan ring), 7.38-7.43 (d, 1H, *J* = 2.5Hz, -CH, furan ring), 8.35 (s, 1H, *-*CH, triazole ring); MS ((*m/z*)/ %) (297, 58); ^13^C-NMR (DMSO-*d_6_*, *δ* / ppm), 21.17, 21.73, 111.53, 116.89, 136.91, 139.78, 142.89, 143.20, 152.96, 153.57, 155.03, 155.72, 168.29; Analytical calculated for C_13_H_11_N_7_O_2_ (297.28): C, 52.52; H, 3.73; N, 32.98; Found: C, 52.40; H, 3.90; N, 32.90 %.

### 8-methyl-5-(5-methylfuran-2-yl)-7-((2,3,4-trimethoxybenzylidene)amino)-pyrimido[5,4-e][1,2,4]triazolo[1,5-a]pyrimidin-6-one (4)

An equimolar mixture of compound **3** (10 mmol) and 2,3,4-trimethoxybenzaldehyde (10 mmol) dissolved in ethanol (10 ml) and a catalytic amount of acetic acid was refluxed for eight hours. The reaction mixture was allowed to cool to room temperature. The precipitate so-formed was filtered off, washed with cold ethanol (1 ml) and recrystallized from ethanol. Pale yellow crystals yield 74 %; mp 201-203 °C. IR (KBr, *ν* / cm^-1^); 1701 (C=O), 1064 (C=C); ^1^H-NMR (DMSO-*d_6_*, *δ* / ppm), 2.36 (s, 3H,CH_3_), 2.38 (s, 3H, CH_3_), 3.39 (s, 3H, -OCH_3_), 3.41 (s, 3H, -OCH_3_), 3.44 (s, 3H, -OCH_3_), 6.58-6.59 (d, 1H, *J* = 2.5Hz, -CH, furan ring), 7.57-7.58 (d, 1H, *J* = 2.5Hz, -CH, furan ring), 7.73-7.78(d, 1H, *J* = 6.7Hz, aromatic proton), 8.12-8.15(d, 1H, *J* = 6.7Hz, aromatic proton), 8.08 (s, 1H, CH, triazole ring), 9.02 (s, 1H, -CH=N); MS ((*m/z*) / %) (475, 68); Analyatical calculatedfor C_23_H_21_N_7_O_5_ (475.47): C, 58.10; H, 4.45; N, 20.62; Found: C, 58.22; H, 4.30; N, 20.88 %.

### Synthesis of in-situ selenium nanoparticles (SeNPs) using the synthesized heterocyclic compound 4

Selenious acid (H_2_SeO_3_, 0.013 g, 0.01 mmol.) was dissolved in 80 ml of deionized water. Compound **4** (0.01 mmol.) in DMSO (10 ml) was added to the solution of selenious acid, then the solution was heated to 60 °C under continuous stirring for 1 h. Afterwards, 200 μL of 40 mM ascorbic acid was added as a catalyst to reduce selenium ions into selenium nanoparticles. The formation of selenium nanoparticles was confirmed and characterized by UV-spectrophotometer, particle size, SEM and TEM.

### Preparation of polycaprolactone nanocapsule

The polycaprolactone nanocapsules were prepared using the emulsion technique, as reported in the literature [25]. Briefly, polycaprolactone (0.25 g) was dissolved in 10 ml water-immiscible organic solvent with a low boiling point as methylene chloride. The triazole derivative was also dissolved in the same solvent in 5 ml and added dropwise to the PCL solution keeping the ratio of triazole to PCL was 10 %. The produced solution was dropped into 30 ml water containing 0.05 % polyvinyl alcohol (PVA) as stabilizing agent under vigorous stirring using a homogenizer at 18.000 rpm for about 30 min. The organic solvent was left to evaporate at room temperature (35 °C) for 24 h under mild stirring to obtain the nanocapsule of PCL containing the triazole derivative. In the case of conjugation of selenium nanoparticles, the selenium nanoparticles were synthesized firstly by reduction of (1 mM) selenium oxide using ascorbic acid (half equimolar ratio) in 0.05 % PVA aqueous solution. The obtained suspension solution was centrifuged at 6000 rpm for about 30 min and the precipitate was freeze-dried.

### Anticancer activity evaluation

#### Cells

**1-Human breast cancer cell line (MCF7)** was obtained from American Type Culture Collection (Rockville, Maryland, USA) and is being maintained in the LudwikHirszfeld Institute of Immunology and Experimental Therapy (Wrocław, Poland). Cells were cultured in Eagle medium (IIET, Wroclaw, Poland) supplemented with 2 mM L-glutamine, 10 % fetal bovine serum, 8 g/mL of insulin and 1 % minimum essential medium (MEM) with non-essential amino acid solution 100e(all from Sigma–Aldrich Chemie GmbH, Steinheim, Germany).

**2-Murine fibroblast normal cell line (BALB/3T3)** was cultured in DMEM (Gibco, UK). It was supplemented with 2 mM L-glutamine, 10 % fetal bovine serum (GE Healthcare, Logan, UT, USA). All culture media were also supplemented with antibiotics. 100 μg/ml streptomycin (Sigma–Aldrich Chemie GmbH, Steinheim, Germany) and 100 units/ml penicillin (PolfaTarchomin SA, Warsaw, Poland). All cell lines were grown at 37 °C with 5 % CO_2_ humidified atmosphere.

#### An in vitro antiproliferative assay

24 hours before adding the tested compounds, the cells were plated in 96-well plates (Sarstedt, Germany) at a density of 10^4^ cells per well. The assay was performed after 72 hours of exposure to varying concentrations of the tested compounds. The *in vitro* cytotoxic effect of all compounds was examined using the SRB assay.

#### Cytotoxic test SRB

The details of this technique were described by Skehan*et al*. [26]. The cells were attached to the bottom of plastic wells by fixing them with cold 50 % TCA (trichloroacetic acid, Sigma-Aldrich Chemie GmbH, Steinheim, Germany) on the top of the culture medium in each well. The plates were incubated at 4 °C for 1 hour and then washed five times with tap water. The cellular material fixed with TCA was stained with 0.4 % sulphorhodamine B (SRB, Sigma-Aldrich Chemie GmbH, Steinheim, Germany) dissolved in 1 % acetic acid (POCH, Gliwice, Poland) for 30 minutes. Unbound dye was removed by rinsing (5 times) in 1 % acetic acid. The protein-bound dye was extracted with 10 mM buffered Tris base (POCH, Gliwice, Poland) for determination of the optical density (*λ* = 540 nm) in Synergy H4 multi-mode microplate reader (BioTek Instruments USA). The relation between surviving fraction and drug concentration is plotted to get the survival curve for each cell line after the specified time. The concentration required for 50 % inhibition of cell viability (*IC*_50_) was calculated and the results are given in Table 1. The results were compared to the antiproliferative effects of the reference control doxorubicin. We used the traditional drug doxorubicin as an independent control in the experiments with *in vitro* antiproliferative activity.

### Statistical analysis

The results are reported as mean ± standard error (SE). The experiments investigating the *in vitro* antiproliferative activity were set in three biological replicates per sample for analysis.

## Results and discussion

**Scheme 1.**
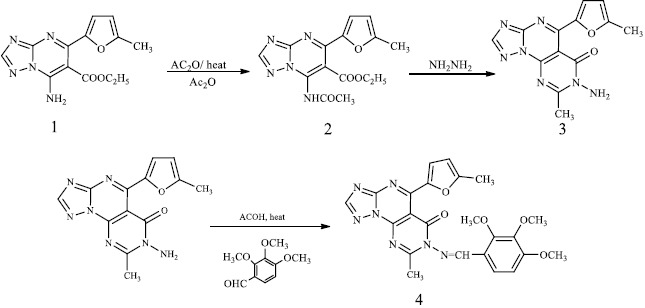
The proposed steps for the synthesis of triazolopyrimidine derivatives.

The synthesis of triazolopyrimidine(s) (**1**) was reported by a simple addition of an equimolecular mixture of amino triazole, carbonyl compounds and active methylene compounds in an aqueous medium [27]. The triazolopyrimidine (**1**) was refluxed in acetic anhydride for 4 hours to produce compound (**2**). The latter showed a new IR peak at 1659 cm^-1^ (C=O), denoting the resulting amide carbonyl with recent signals for ^1^H-NMR (DMSO-*d_6_*, *δ*/ ppm) and 2.33 (s, 3H, CH_3_). Triazolopyrimidine (**2**) treated with hydrazine hydrate under reflux formed compound (**3**) possessing a new IR peak at 1669 cm^-1^ (C=O), with the disappearance of the acetate carbonyl group. The synthesis of triazolopyrimidine derivative (**4**) was carried out by the reaction of amino triazole (**3**) with trimethoxybenzaldehyde in the presence of acetic acid to have new signals for the new three methoxy groups and signals for the new aromatic protons ^1^H-NMR (DMSO-*d_6_*, *δ* / ppm), 3.39 (s, 3H, -OCH_3_), 3.41 (s, 3H, -OCH_3_), 3.44 (s, 3H, -OCH_3_), 7.73-7.78 (d, 1H, *J* = 6.7 Hz, aromatic proton), 8.12-8.15 (d, 1H, *J* = 6.7 Hz, aromatic proton) as shown in Scheme 1.

### Synthesis of in-situ selenium nanoparticles using heterocyclic compounds (4) (HET-SeNPs)

**Figure 1.**
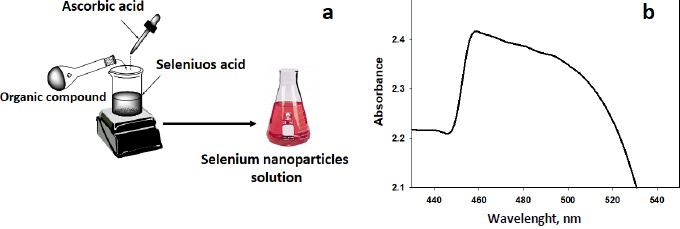
(a) The pathway for the synthesis of SeNPs, (b) UV-Vis spectrum of SeNPs.

The synthesis of selenium nanoparticles (SeNPs) conjugated with the synthesized compound (**4**) was performed. The triazole derivative has an adequate balance of low reducing and stabilizing properties of nanoparticles. Ascorbic acid was utilized as a catalyst. It is employed to generate SeNPsby stabilizing their nanostructure upon reducing the Se^+^ cation into Se [28-31]. Some organic compounds with reductive groups, such as -OH, -SH, -NH, can reduce selenium cation. The pathway to synthesize SeNPs is depicted in Figure 1a. UV-VIS spectroscopy was used to monitor the formation of SeNPs, as illustrated in Figure 1b. According to the surface plasmon of selenium colloidal solution, a resonance peak appears in the absorption spectrum at 465 nm, referring to SeNPs.

**Figure 2.**
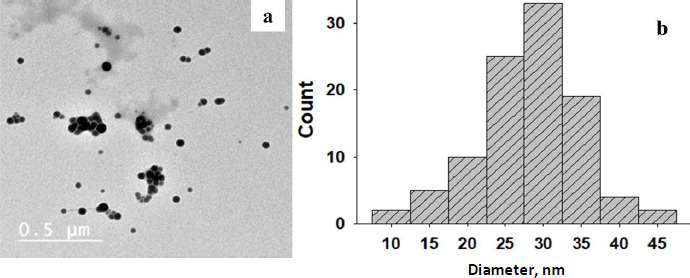
TEM of SeNPs and their diameter distribution histogram from image analysis.

The formation of selenium nanoparticles was confirmed by TEM analysis [32]. In Figure 2a, SeNPsare displayed as spheres with few agglomerates in a global micrograph.

The diameter distribution of these nanoparticles ranges between 25-34 nm, as displayed in Figure 2b. The diameter distribution of these nanoparticles was assessed through ImageJ software in the experimental section. The nanoparticles seem to be apart in an acceptable allocation [33,34].

**Figure 3.**
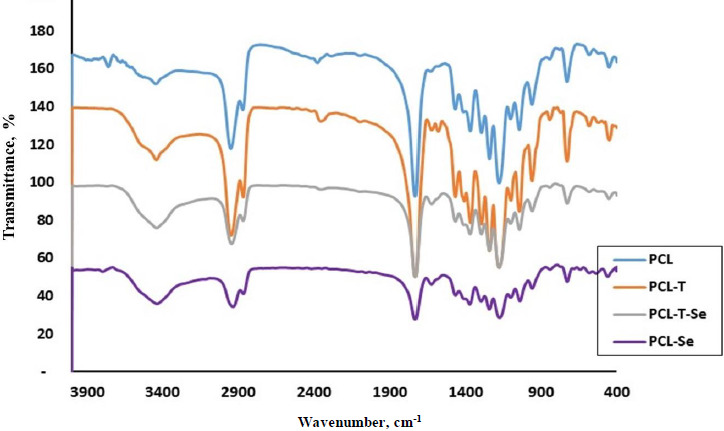
FTIR of PCL, PCL-triazole, PCL-triazole-Se and PCL-Se nanoparticles.

The reactive functional groups of PCL and its encapsulated nanoparticles are shown in Figure 3. Polycaprolactone shows a strong peak at 1736 cm^-1^ of carbonyl C=O groups. The peak at 1185 corresponds to C-O stretching band. CH symmetrical and asymmetrical vibrations appear at 2868 and 2947 cm^–1^, respectively. For triazole-encapsulated PCL, there is a small peak at 1640 cm^–1^ related to C=N of heterocyclic structure. In addition, the peak at 3445 cm^–1^ indicates O-H or N-H stretching bands.

**Figure 4.**
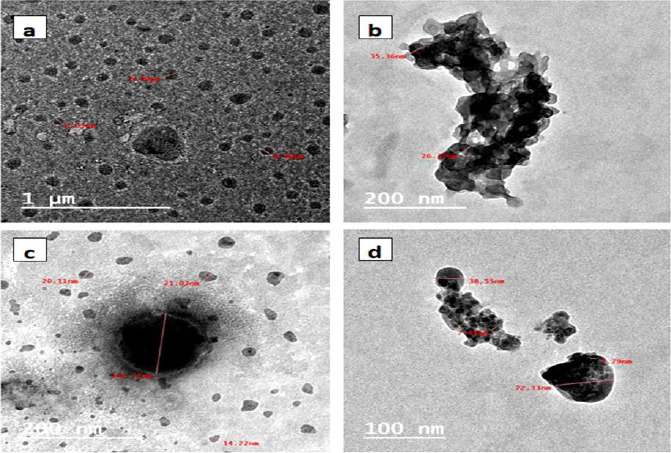
TEM of (a) PCL, (b) PCL-triazole, (c) PCL-triazole- Se and (d) PCL-Se nanoparticles.

The size and shape of the prepared polycaprolacone nanoparticles were evaluated using TEM, as shown in Figure 4. One can see in Figure 4a that the formed blank PCL was in semi-spherical shape with a major average size of 50 to 90 nm. Figure 4b displayed PCL encapsulated triazole derivative with two distinct phases in black and grey color, indicating encapsulated particles and associated with some aggregates. Figure 4c indicates the encapsulated triazole with selenium nanoparticles emerged as semi-spherical particles demonstrating black spots in the middle of each particle. Their sizes varied from 14 to 144 nm, with the major size around 25 nm. Figure 4d showed PCL encapsulated selenium nanoparticles with obvious selenium nanoparticles embedded in spherical polymer particles in the range of 38 to 72 nm.

**Figure 5.**
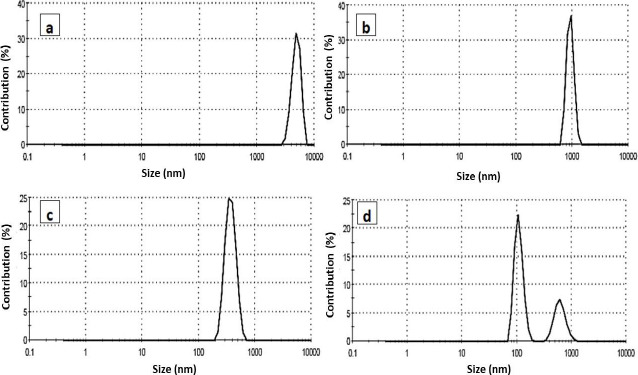
Particle size distribution from DLS for (a) PCL, (b) PCL-triazole, (C) PCL-triazole-Se and (d) PCL-Se nanoparticles.

The particle size distribution of the prepared PCL nanoparticles was explored using dynamic light scattering measurement. The measurements of DLS based on the scattered beam resulted from the movement of the nanoparticle in the suspension solution. The particle size of the PCL and its encapsulated compounds are sown in Figure 5. The size of the blank PCL was large (48 lm), indicated as aggregated particles. The encapsulated PCL was around 980 nm and selenium conjugated triazole was 370 nm for selenium PCL, showing two peaks; the major at 110 nm and the minor at 600 nm. Larger particle sizes were obtained from DLS than in TEM. This feature may be attributed to the fact that, in TEM, the image revealed a small selected part of the dried sample of the nanoparticles, while in DLS, it gives a complete overview of the whole sample, which may be concentrated or aggregated and swelled particles.

### Anticancer performance (antiproliferative activity)

The in vitro antiproliferative activity of the encapsulated polycaprolactone samples was investigated towards human breast cancer (MCF7) and murine fibroblast normal (BALB/3T3) cell lines via standard established assays. The IC_50_ value is defined as the concentration of a compound at which 50 % growth inhibition is observed. The selectivity index (SI) was calculated for each compound using the equation (1):


(1)

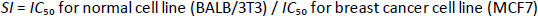



**Figure 6.**
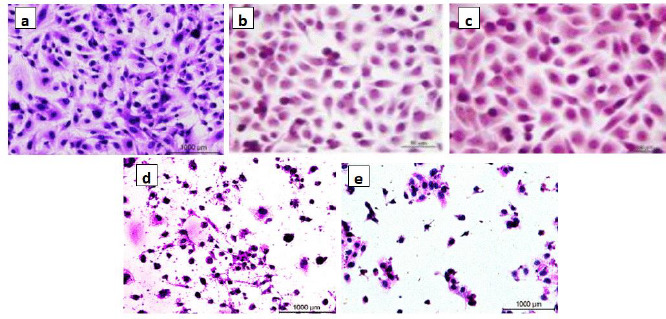
Antiproliferative activity of the encapsulated polycaprolactone towards human breast cancer cell line (MCF7): (a) Untreated, (b) polycaprolactone **1**, (c) encapsulated polycaprolactone + triazole **2**, (d) encapsulated polycaprolactone + triazole-SeNPs **3** and (e) encapsulated polycaprolactone + SeNPs **4**.

**Figure 7.**
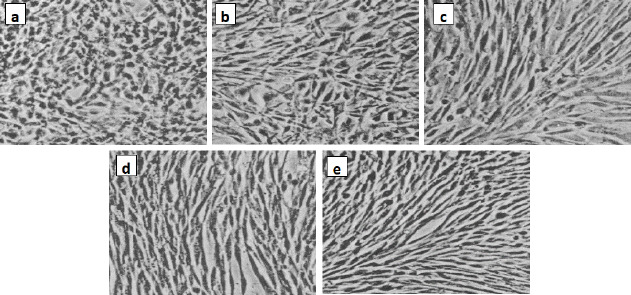
Antiproliferative activity of the encapsulated polycaprolactone towards murine fibroblast normal cell line (BALB/3T3): (a) Untreated, (b) polycaprolactone1, (c) encapsulated polycaprolactone + triazole **2**, (d) encapsulated polycaprolactone + triazole-SeNPs **3** and (e) encapsulated polycaprolactone + SeNPs **4**.

A beneficial SI>1.0 indicates that the drug with efficacy against tumour cells is greater than the toxicity against normal cells. Both the tumour and normal cell lines showed normal growth in our culture system. DMSO did not seem to have any noticeable effect on cellular growth. A gradual decrease in the viability of cancer cells was observed with increasing concentration of the tested compounds, in a dose-dependent inhibitory effect. The data in Table 1, in addition to Figures 6 and 7, indicate that two of the obtained encapsulated capsules (**3** and **4**) were shown to be active against the investigated human breast cancer cell line (MCF-7) with strong potency at lower concentrations and a higher relative affinity to tumour cells over normal cells. Moreover, the results indicated that the salectivity index (SI) was higher than 1.0. Hence, we have newly synthesized compounds with higher antiproliferative efficacy against tumour cells. This potency is greater than the toxicity against normal cells. So, it can be concluded that newly synthesized compounds showed a significant potency towards tested cancer cell lines. However, they displayed weak or moderate activities towards normal cells (BALB/3T3).

### Regression equation

To determine the regression between the *IC*_50_ values for the tested two types of cells, we will estimate the correlation coefficient (*r*), which is used to measure the strength of the relationship between the two *IC*_50_ values using equation (2):


(2)

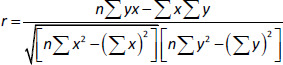



where: *r* = correlation coefficient, *n*= number of samples, =*x* = total of all *IC*_50_ values for breast cancer cells, b*y* = total of all *IC*_50_ values for murine fibroblast normal cells, xy= sum of the product of first and second values, u*x*^2^=sum of squares of the first value, =y^2^=sum of squares of the second value. The calculation of *r* is presented in equation (3):


(3)

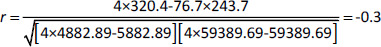



The present result is the product-moment correlation coefficient (or Pearsoncorrelation coefficient). It is known that the value of *r* always lies between –1 and +1. A value of the correlation coefficient close to +1 indicates a strong positive linear relationship (*i.e.*, one variable increases with the other). A value close to -1 indicates a strong negative linear relationship (*i.e.*, one variable decreases as the other increases). A value close to 0 indicates no linear relationship; however, there could be a nonlinear relationship between the variables. For the regression between the *IC*_50_ values of the tested two types of cells presented in Table 1, the correlation coefficient = -0.3, indicates a moderate negative linear relationship between the two variables. As shown in Figure 7, the antiproliferative activity of the encapsulated polycaprolactone compounds (**1**, **2**, **3** and **4**) towards the murine fibroblast normal cell line (BALB/3T3) can be compared with the activities of these compounds towards tested cancer cell line along with doxorubicin as the traditional anticancer drug [35].

## Conclusion

Selenium nanoparticles were prepared and conjugated with triazole derivatives, then encapsulated into polycaprolactone-forming nanocapsules. These synthesized nanocapsules were investigated to explore their antiproliferative and anticancer activities. The tested nanocapsules (**3** and **4**) samples exert significant antiproliferative potency towards the human breast cancer cell line (MCF7) by reducing cell proliferation, resulting in a reasonable significant growth inhibitory effect. On the other hand, all tested compounds gave weak or moderate activities towards normal cells (BALB/3T3). So, the present study reveals that human breast cancer cells (MCF7) are more sensitive to the tested compounds than normal cells (BALB/3T3). These findings indicate a specific antiproliferative potency of these newly synthesized compounds towards cancer cell lines. Further pharmacological investigations are needed to study the efficacy of these compounds.

## Figures and Tables

**Table 1.** *In vitro* antiproliferative activity (IC_50_) of the encapsulated polycaprolactone towards human breast cancer (MCF7) and murine fibroblast normal (BALB/3T3) cell lines and the calculated values of the selectivity index (SI) of **1 -** tested encapsulated polycaprolactone, **2 -** encapsulated polycaprolactone with triazole, **3 -** encapsulated polycaprolactone with triazole-SeNPs and **4 -** encapsulated polycaprolactone with SeNPs.

Compounds	*IC*_50_ ± SD / μg ml^-1^		Selectivity index
	Human breast cancer (MCF7)	Murine fibroblast normal (BALB/3T3)	
1	35.8 ± 6.9	83.5 ± 12.3	2.4
2	24.6 ± 3.4	76.8 ± 10.4	3.1
3	7.2 ± 0.4	38.1 ± 9.2	5.3
4	9.1 ± 0.3	45.3 ± 10.2	4.9
Doxorubicin	5.03 ± 0.7	3.8 ± 0.2	0.75
DMSO	N.A.	N.A.	N.A.

Data were expressed as mean ± SD of three independent experiments. *IC*_50_: 1 to 10 μg ml^-1^ - very strong, 11 to 25 μg ml^-1^ strong, 26 to 50 μg ml^-1^ -moderate; 51 to 100 μg ml^-1^ – weak; DOX: Doxorubicin is the drug reference; N.A.: No activity
